# The Imatinib–miR-335-5p–ARHGAP18 Axis Attenuates PDGF-Driven Pathological Responses in Pulmonary Artery Smooth Muscle Cells

**DOI:** 10.3390/ijms26199368

**Published:** 2025-09-25

**Authors:** Yunyeong Lee, Hara Kang

**Affiliations:** 1 Division of Life Sciences, College of Life Sciences and Bioengineering, Incheon National University, Incheon 22012, Republic of Korea; yuneongii@inu.ac.kr; 2 Institute for New Drug Development, Incheon National University, Incheon 22012, Republic of Korea

**Keywords:** imatinib, microRNA, PDGF signaling, pulmonary artery smooth muscle cell

## Abstract

The proliferation and migration of pulmonary artery smooth muscle cells (PASMCs) are key pathological features of vascular remodeling during pulmonary hypertension. Platelet-derived growth factor (PDGF) signaling is a major contributor to these processes. Given the importance of microRNA (miRNA) regulation in the PDGF signaling pathway in PASMCs, we hypothesized that imatinib, a tyrosine kinase inhibitor, modulates the expression levels of miRNAs responsive to PDGF signaling to ameliorate the PDGF signaling-induced PASMC phenotype. In this study, we investigated the role of miR-335-5p in PDGF signaling-induced PASMC proliferation and migration, as well as the involvement of imatinib in the regulatory network of miR-335-5p. miR-335-5p was identified as a critical negative regulator of PDGF signaling. Functional assays revealed that miR-335-5p significantly inhibits PASMC proliferation and migration. Through target prediction and validation, Rho GTPase Activating Protein 18 (ARHGAP18) was identified as a novel direct target of miR-335-5p. In addition, ARHGAP18 was found to play an essential role in regulating PASMC proliferation and migration. Although miR-335-5p was downregulated upon PDGF-BB stimulation, its expression was restored by imatinib. These findings highlight the important role of the imatinib–miR-335-5p–ARHGAP18 axis as a potential therapeutic target for pathological vascular remodeling.

## 1. Introduction

Platelet-derived growth factor (PDGF) is a strong mitogen for pulmonary artery smooth muscle cells (PASMCs) [1,2]. Activation of the PDGF signaling pathway by binding to its receptors PDGFRα and PDGFRβ leads to phosphorylation of downstream molecules, including Akt, and promotes the synthetic phenotype by suppressing contractile gene expression and stimulating the proliferation and migration of PASMCs [3]. Previous studies have reported that abnormal increase in proliferation and migration of PASMCs are implicated in the development and progression of pulmonary vascular remodeling in pulmonary arterial hypertension (PAH) [4]. PAH is a complex disorder characterized by vascular remodeling and a consequent increase in pulmonary vascular resistance [5]. PDGF and PDGFR are upregulated in PAH and inhibition of PDGFR signaling prevents the development of PAH in animal models and reverses pulmonary vascular remodeling [6]. Therefore, the PDGF signaling pathway has been investigated as a potential therapeutic target for PAH [7]. Imatinib is a tyrosine kinase inhibitor initially developed to treat chronic myeloid leukemia (CML), and it inhibits Bcr-Abl, c-KIT, and PDGFR [8]. Imatinib prevents phosphorylation of PDGFR, thereby suppressing activation of the downstream signaling pathways associated with PAH. As an inhibitor of PDGFR, imatinib has been shown to inhibit the proliferation and migration of PASMCs, and reverse pulmonary vascular remodeling in PAH in rodents and humans [9,10]. Recent clinical trials with imatinib showed reduced pulmonary vascular resistance, but systemic side effects limit its further development [4]. Despite adverse events, it remains an interesting target in pulmonary vascular remodeling in PAH.

MicroRNAs (miRNAs) control gene expression by destabilizing targeted transcripts and inhibiting their translation, affecting many biological processes, including cell differentiation, survival, and proliferation [11,12]. The aberrant expression of miRNAs has been described in many human diseases, including cancer and cardiovascular diseases [13]. Many miRNAs have been identified as critical mediators for the modulation of the vascular smooth muscle cell phenotype in response to extracellular signals, including PDGF. The PDGF signaling-specific signature of miRNAs indicates that the epigenetic dysregulation of miRNAs is involved in PAH [14,15,16]. Although the effect of imatinib on miRNA expression in PAH has not been thoroughly examined, imatinib affects the characteristic miRNA expression profile in patients with CML [17,18]. Several miRNAs can regulate the effectiveness of imatinib or contribute to the acquired imatinib resistance in CML [19,20,21,22,23]. Therefore, it is interesting to investigate the regulation of miRNA upon imatinib treatment in PAH.

miR-335-5p is differentially expressed in a variety of human cancer cells, including breast and gastric cancer cells [24,25]. miR-335-5p serves as a suppressor of cancer cell migration, invasion, and metastasis by targeting several genes, including SP1, ROCK1, and MAPK1 [24,25]. Moreover, miR-335-5p is highly expressed in human mesenchymal stem cells and is downregulated upon differentiation, orchestrating cell proliferation, migration and differentiation [26]. Although the function and potential mechanism of miR-335-5p in the pathogenesis of PAH have not yet been thoroughly examined, a recent study has shown that miR-335-5p regulates proliferation and migration in human aortic vascular smooth muscle cells [27]. Therefore, whether miR-335-5p plays a role in the regulation of PASMC functions remains to be elucidated.

In this study, we hypothesized that imatinib regulates the miRNA-mediated epigenetic regulation of gene expression in PDGF signaling-stimulated PASMCs. In investigating the effect of imatinib on the miRNA-mediated regulation of the PASMC phenotype, we examined the functional relevance of imatinib–miR-335-5p in the responses of PASMCs to PDGF signaling, including proliferation and migration.

## 2. Results

### 2.1. Imatinib Inhibits PDGF Signaling-Induced Proliferation and Migration of PASMCs

**Figure 1:**
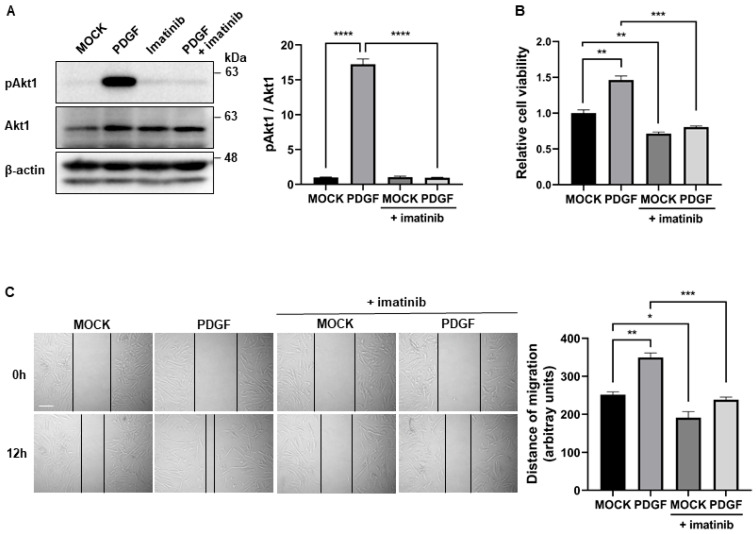
Imatinib inhibits PDGF signaling-induced proliferation and migration of PASMCs. (**A**) Immunoblot analysis of phosphorylated Akt1 (pAkt1), total Akt1, and β-actin in PASMCs treated for 1 h with vehicle (MOCK), PDGF-BB (40 ng/mL), imatinib (5 μM), or a combination of both. Densitometric analysis was used to quantify pAkt1 levels normalized to total Akt1. Data represent the mean ± SE of triplicates and were analyzed using one-way ANOVA followed by Tukey’s multiple comparisons test. (**B**) CCK-8 assay showing the relative absorbance (OD) values of viable PASMCs treated for 3 days with PDGF-BB, imatinib, or both. Data represent the mean ± SE of triplicates and were analyzed by one-way ANOVA with Tukey’s test. (**C**) Scratch wound assay of PASMCs treated as above. Migration distances were measured using ImageJ at 12 h post-wounding. Representative images (**left**) and quantification graphs (**right**) are shown. Black lines indicate the measured distance of cell migration into the scratched area. Data represent the mean ± SE of triplicates from three independent experiments. One-way ANOVA with Tukey’s test was used for statistical analysis. *, *p* < 0.05; **, *p* < 0.005; ***, *p* < 0.0005; ****, *p* < 0.0001. Scale bar: 100 µm.

In ascertaining whether imatinib inhibits PDGF signaling in PASMCs, cells were treated with 40 ng/mL PDGF-BB for 1 h in the absence or presence of 5 µM imatinib. The phosphorylation level of Akt, which is a downstream effector of PDGF signaling, was analyzed by immunoblotting. As expected, imatinib inhibited PDGF-induced Akt phosphorylation (Figure 1A). Then, the effects of imatinib on the function of PASMCs were investigated by examining their proliferation and migratory activity. The cell counting kit-8 (CCK-8) assay was used for the spectrophotometric quantification of cell proliferation. PASMCs were treated with PDGF-BB with or without imatinib for 72 h. The increased cell proliferation in response to PDGF-BB was reduced by imatinib treatment (Figure 1B). In determining the effects of imatinib on migration, an in vitro scratch wound assay was performed. Scratch wounds were created in PASMCs treated with PDGF-BB in the absence or presence of imatinib. Wound closure was monitored over time. We found that the accelerated rate of cell migration induced by PDGF-BB was delayed by imatinib treatment (Figure 1C). Together, these results indicate that imatinib inhibits the PDGF signaling-induced proliferation and migration of PASMCs.

### 2.2. Imatinib Affects the Expression of miRNAs That Respond to PDGF Signaling

**Figure 2:**
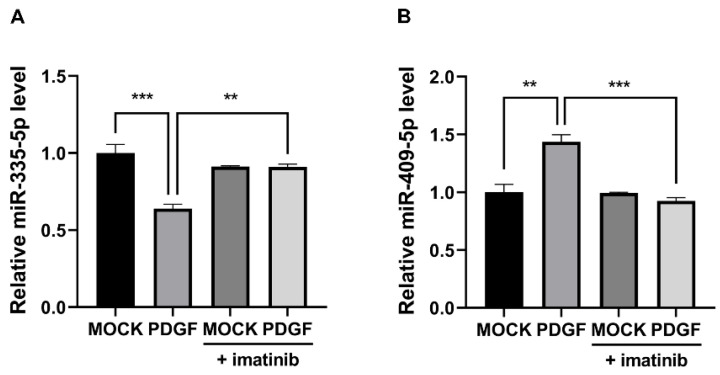
Imatinib affects the expression of miRNAs that respond to PDGF signaling. Relative expression levels of miR-335-5p (**A**) and miR-409-5p (**B**) normalized to U6 snRNA in PASMCs treated with PDGF-BB, imatinib, or both, measured by qRT-PCR. Data represent the mean ± SE of triplicates and were analyzed using one-way ANOVA with Tukey’s test. **, *p* < 0.005; ***, *p* < 0.0005.

Considering that PDGF signaling regulates PASMCs functions through miRNAs, we investigated whether imatinib modulates PDGF signaling-induced changes in cell function via miRNA regulation. We first tested whether the expression levels of miRNAs, such as miR-335-5p and miR-409-5p, known to be altered by PDGF signaling, were affected by imatinib [27,28]. A recent study has reported the function of miR-335-5p in association with PDGF signaling in vascular cells, and our previous research identified miR-409-5p as a key miRNA in PDGF signaling-induced vascular pathogenesis. PASMCs were treated with PDGF-BB for 24 h with or without imatinib, and then the miRNA levels were measured by qRT–PCR. Interestingly, the changes in the expression levels of these miRNAs induced by PDGF signaling were reversed by imatinib. The expression level of miR-335-5p decreases upon PDGF-BB stimulation, but cotreatment with imatinib restores the reduced expression level of miR-335-5p induced by PDGF signaling (Figure 2A). In contrast, the expression level of miR-409-5p, which increases upon PDGF-BB stimulation, decreases to the untreated control level (mock) when cotreated with imatinib (Figure 2B). These results suggest that imatinib broadly modulates PDGF-regulated miRNA expression in PASMCs, thereby preventing PDGF-induced alterations in cellular functions. Although imatinib modulated the expression of both miR-335-5p and miR-409-5p in response to PDGF signaling, the present study was designed to focus on the mechanistic role of the miR-335-5p–ARHGAP18 axis, which we identified as functionally critical in PASMC regulation.

### 2.3. miR-335-5p Inhibits the Activation of PDGF Signaling

**Figure 3:**
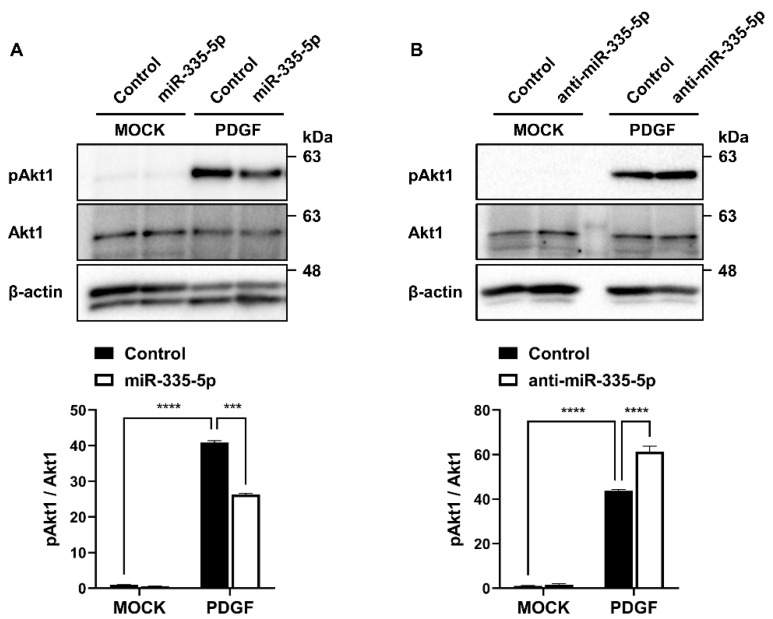
miR-335-5p inhibits the activation of PDGF signaling. (**A**,**B**) Immunoblot analysis of pAkt1, Akt1, and β-actin in PASMCs transfected with miR-335-5p mimic (**A**) or anti-miR-335-5p (**B**), followed by PDGF-BB stimulation. Densitometric quantification of pAkt1 levels normalized to total Akt1 is shown. Data represent the mean ± SE of triplicates and were analyzed by two-way ANOVA with Tukey’s multiple comparisons test. ***, *p* < 0.0005; ****, *p* < 0.0001.

In investigating the functional relationship between miR-335-5p and PDGF signaling in PASMCs, the effect of miR-335-5p on the activation of PDGF signaling was examined. Cells were transfected with miR-335-5p mimic, miR-335-5p antisense inhibitor RNA (anti-miR-335-5p), or control mimic for 24 h and then stimulated with PDGF-BB. In cells transfected with the miR-335-5p mimic, Akt phosphorylation induced by PDGF-BB stimulation was reduced (Figure 3A). In contrast, in cells transfected with anti-miR-335-5p, Akt phosphorylation induced by PDGF-BB stimulation was further increased (Figure 3B). These results indicate that miR-335-5p negatively regulates PDGF signaling. Thus, PDGF-BB may facilitate the PDGF signaling pathway by downregulating miR-335-5p.

### 2.4. miR-335-5p Inhibits the Proliferation and Migration of PASMCs

**Figure 4:**
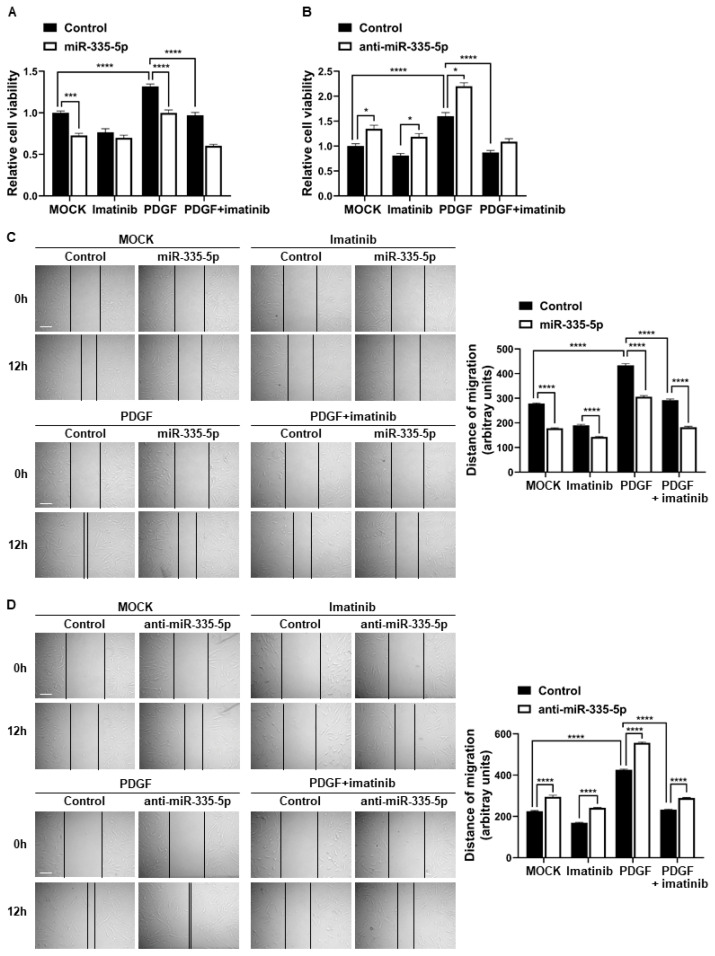
miR-335-5p inhibits the proliferation and migration of PASMCs. (**A**,**B**) CCK-8 assay of PASMCs transfected with miR-335-5p mimic (**A**) or anti-miR-335-5p (**B**) and subsequently treated with PDGF-BB and/or imatinib. (**C**,**D**) Scratch wound assay of PASMCs transfected as above and treated with PDGF-BB and/or imatinib. Migration distances were measured using ImageJ at 12 h post-wounding. Representative images (**left**) and quantification graphs (**right**) are shown. Black lines indicate the measured distance of cell migration into the scratched area. Data represent the mean ± SE of triplicates from three independent experiments. Statistical analysis was performed using two-way ANOVA with Tukey’s test. *, *p* < 0.05; ***, *p* < 0.0005; ****, *p* < 0.0001. Scale bar: 100 µm.

To investigate the significance of miR-335-5p on the regulation of PASMC functions, we examined whether miR-335-5p affects the proliferation of PASMCs. PASMCs were transfected with negative control miRNA, miR-335-5p mimic, or anti-miR-335-5p for 96 h and then subjected to a cell proliferation assay using the CCK-8 assay kit. The cell proliferation rate in cells transfected with miR-335-5p was reduced by 27.2%, which is similar to that in cells treated with imatinib (Figure 4A). By contrast, the cell proliferation rate in cells transfected with anti-miR-335-5p was enhanced by 1.35-fold (Figure 4B). After miR-335-5p transfection into the cells followed by PDGF-BB treatment, the increase in cell proliferation induced by PDGF signaling was inhibited (Figure 4A). In contrast, anti-miR-335-5p transfection, followed by PDGF-BB treatment, resulted in a further enhancement of cell proliferation compared with PDGF-BB stimulation alone (Figure 4B). These results indicate that miR-335-5p inhibits PASMC proliferation.

Subsequently, we examined the effects of miR-335-5p on the migratory activity of PASMCs. Cells were transfected with negative control miRNA, miR-335-5p mimic, or anti-miR-335-5p for 24 h and then subjected to an in vitro scratch wound assay. The migration distance in cells transfected with the miR-335-5p mimic was reduced by 36% compared with that is cells transfected with the negative control miRNA, which is similar to the migration distance in cells treated with imatinib. In addition, the increase in cell migration rate induced by PDGF-BB stimulation was inhibited by miR-335-5p overexpression (Figure 4C). In contrast, when miR-335-5p expression was downregulated using anti-miR-335-5p, the cell migration rate increased by 1.3-fold compared with control cells, and the PDGF signaling-induced increase in migration was further enhanced (Figure 4D). These results indicate that miR-335-5p inhibits the migratory activity of PASMCs. Taken together, miR-335-5p inhibits the proliferation and migration of PASMCs, indicating that the PDGF-induced downregulation of miR-335-5p plays a pivotal role in regulating PASMC functions through the PDGF signaling pathway.

Moreover, the effect of imatinib on reducing PDGF signaling-induced cell migration was enhanced when miR-335-5p was overexpressed (Figure 4C) and diminished when miR-335-5p was downregulated (Figure 4D). Furthermore, when miR-335-5p was downregulated, cell proliferation increased by 46.6% even after imatinib treatment (Figure 4B). These results indicate that the expression level of miR-335-5p may influence the function of imatinib. PDGF signaling decreases the levels of miR-335-5p that inhibit the proliferation and migration of PASMCs, whereas imatinib appears to restore miR-335-5p expression, thereby recovering the changes in cellular function induced by PDGF signaling.

### 2.5. ARHGAP18 Is a Novel Target of miR-335-5p

**Figure 5:**
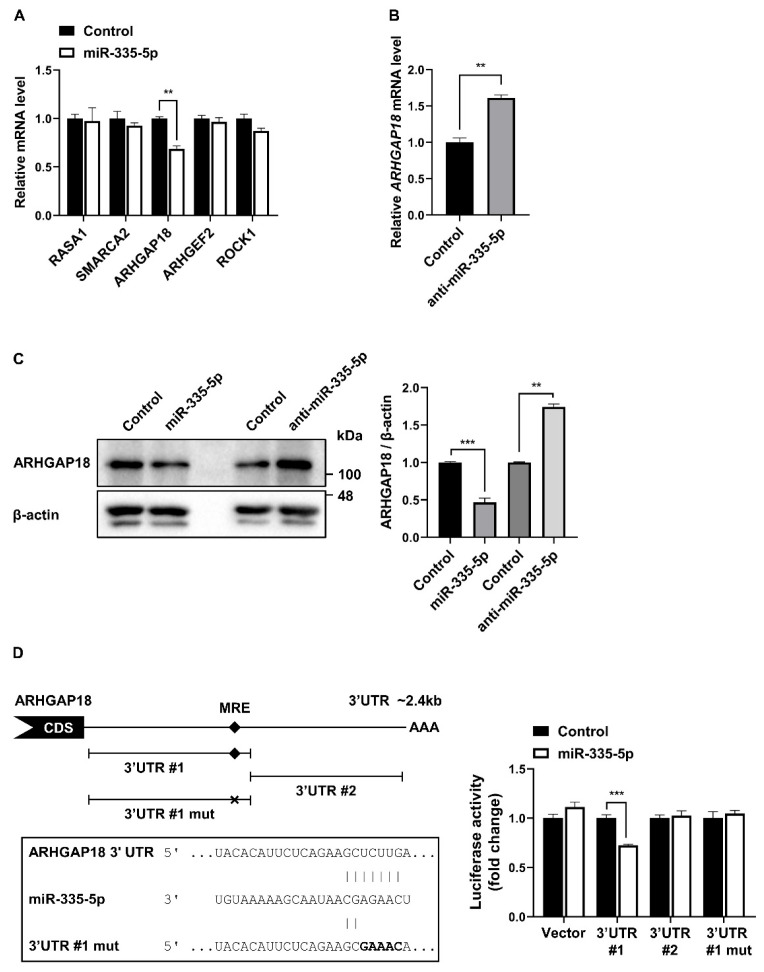
ARHGAP18 is a novel target of miR-335-5p. (**A**) qRT-PCR analysis of the indicated mRNAs relative to *GAPDH* in PASMCs transfected with miR-335-5p mimic. Two-way ANOVA with Sidak’s multiple comparisons test was used. (**B**) *ARHGAP18* mRNA levels relative to 18S rRNA in PASMCs transfected with anti-miR-335-5p. Two-tailed unpaired t-test was used. (**C**) Immunoblot analysis of ARHGAP18 and β-actin in PASMCs transfected with control, miR-335-5p mimic, or anti-miR-335-5p. Protein expression was quantified by densitometry and normalized to β-actin. (**D**) (**Left panel**) Schematic diagram of luciferase reporter constructs used for luciferase assays and predicted miR-335-5p MRE in the 3′UTR of ARHGAP18 transcripts. CDS and AAA stand for protein coding sequence and poly(A) tail, respectively. Wildtype MRE and mutant MRE disrupted a base pairing with miR-335-5p sequence are indicated as black diamond and X, respectively. The sequences of the 3′UTR and the 3′UTR mutant cloned downstream of the luciferase gene are shown. The mutated sequence is in bold. Perfect base matches are indicated by a line. (**Right panel**) Luciferase activity of constructs was assessed in Cos7 cells following transfection with either a control or miR-335-5p mimic. A luciferase vector without 3′UTR sequence (Vector) was used as a negative control. Data are presented as the mean ± S.E. of triplicates. **, *p* < 0.005; ***, *p* < 0.0005.

To understand the molecular mechanism of miR-335-5p through its target mRNA, we searched for potential target mRNAs using the TargetScan target prediction algorithm and the miRWalk database. From these results, we selected genes associated with cell proliferation and migration, including RAS P21 Protein Activator 1 (*RASA1*), SWI/SNF Related, Matrix Associated, Actin Dependent Regulator of Chromatin, Subfamily A, Member 2 (*SMARCA2*), Rho GTPase Activating Protein 18 (*ARHGAP18*), Rho/Rac Guanine Nucleotide Exchange Factor 2 (*ARHGEF2*), and Rho Associated Coiled-Coil Containing Protein Kinase 1 (*ROCK1*). Among the genes tested, qRT-PCR results showed that the level of *ARHGAP18* mRNA relative to *GAPDH* was reduced to 68.5% in miR-335-5p mimic-transfected PASMCs compared with control miRNA-transfected PASMCs (Figure 5A). Conversely, the mRNA level of *ARHGAP18* increased 1.6-fold when miR-335-5p was downregulated using anti-miR-335-5p (Figure 5B). These findings suggest that miR-335-5p suppresses the expression of *ARHGAP18*. We then examined the expression level of ARHGAP18 protein using Western blotting. Immunoblot analysis showed that the endogenous ARHGAP18 protein level in PASMCs was reduced by the exogenous miR-335-5p mimic. In contrast, the basal level of ARHGAP18 protein was elevated by anti-miR-335-5p in comparison with the control (Figure 5C). These results indicate that ARHGAP18 is a novel target of miR-335-5p.

ARHGAP18 as a target of miR-335-5p was further validated using a luciferase assay (Figure 5D). Given that only one miRNA response element (MRE) of miR-335-5p in the full length of ARHGAP18 3′UTR was predicted by computer algorithms, two constructs containing parts of the ARHGAP18 3′UTR were generated: one including the predicted MRE (3′UTR #1) and the other without it (3′UTR #2). The luciferase activity of the 3′UTR construct including MRE, was reduced upon overexpression of the miR-335-5p mimic, but not that of the 3′UTR construct without the MRE. In addition, the luciferase activity of the 3′UTR mutant construct, which is the same as the 3′UTR #1 construct except for a mutated MRE region (3′UTR #1 mut), was not changed by the miR-335-5p mimic. These results indicate that miR-335-5p suppresses the expression of ARHGAP18 by directly binding to the MRE within the 3′UTR.

### 2.6. ARHGAP18 Regulates the Proliferation and Migration of PASMCs

**Figure 6:**
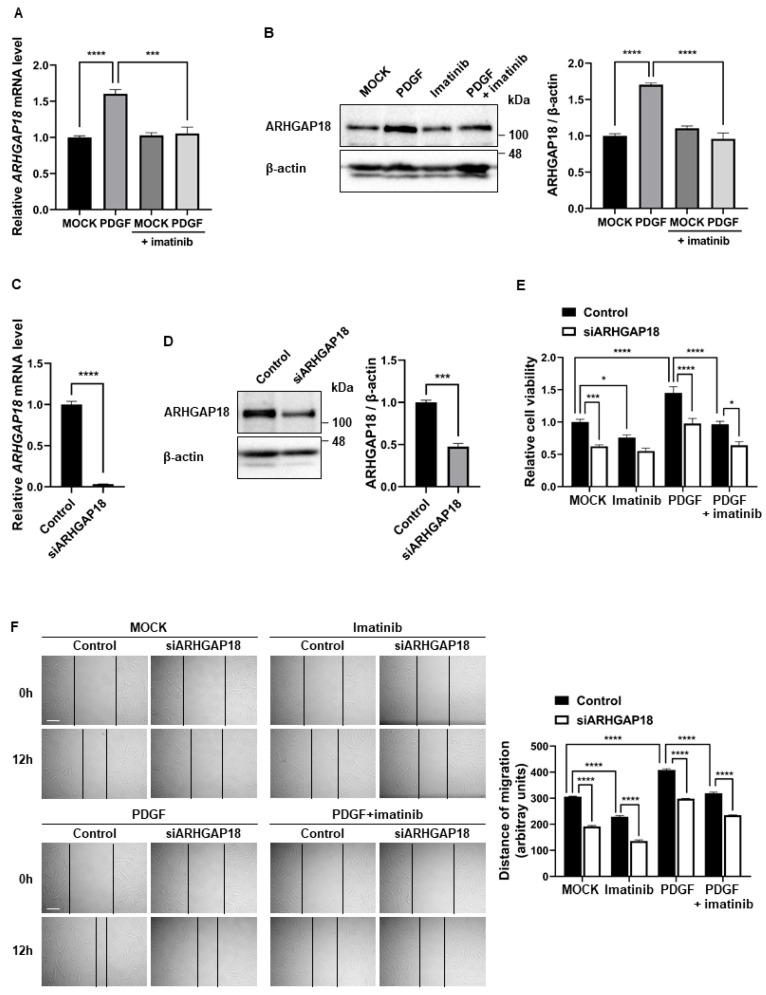
ARHGAP18 regulates the proliferation and migration of PASMCs. (**A**) qRT-PCR analysis of *ARHGAP18* mRNA relative to 18S rRNA in PASMCs treated with PDGF-BB and/or imatinib for 24 h. (**B**) Immunoblot analysis of ARHGAP18 and β-actin under the same conditions, with densitometric quantification. (**C**,**D**) qRT-PCR (**C**) and immunoblot (**D**) analysis of ARHGAP18 in PASMCs transfected with siARHGAP18 or control. (**E**) CCK-8 assay of PASMCs transfected with siARHGAP18 for 24 h, followed by PDGF-BB and/or imatinib treatment. (**F**) Scratch wound assay of PASMCs transfected with siARHGAP18 for 48 h and treated as indicated. Migration distances were measured at 12 h post-wounding. Representative images (**left**) and quantification graphs (**right**) are shown. Black lines indicate the measured distance of cell migration into the scratched area. Data represent the mean ± SE of triplicates from three independent experiments. One-way or two-way ANOVA with Tukey’s test was used. *, *p* < 0.05; ***, *p* < 0.0005; ****, *p* < 0.0001. Scale bar: 100 µm.

As PDGF signaling reduced the expression level of miR-335-5p, we examined the effects of PDGF signaling on ARHGAP18 expression using qRT-PCR and Western blotting. The mRNA and protein levels of ARHGAP18 increased in PASMCs stimulated with PDGF-BB, and this increase was inhibited when cells were co-treated with imatinib (Figure 6A,B). These results indicate that the downregulation of miR-335-5p by PDGF signaling leads to the derepression of ARHGAP18 expression, and that the imatinib-mediated regulation of miR-335-5p level affects ARHGAP18 expression.

Next, we investigated whether ARHGAP18 affects PASMC phenotypes because miR-335-5p inhibits PASMC proliferation and migration. The effects of ARHGAP18 expression on PASMC proliferation were assessed by downregulating ARHGAP18 using siRNA. The knockdown efficiency of siRNA targeting ARHGAP18 (siARHGAP18) was confirmed by qRT-PCR and immunoblotting (Figure 6C,D). ARHGAP18 mRNA and protein levels were reduced by siRNAs by 96.8% and 52.4%, respectively. PASMCs transfected with siARHGAP18 or negative control siRNA (control) were subjected to a cell proliferation assay using the CCK-8 assay kit (Figure 6E). In siARHGAP18-transfected PASMCs, cell proliferation decreased by 37.6% compared with control cells. Moreover, the increase in cell proliferation induced by PDGF signaling was inhibited in ARHGAP18-knockdown cells. These results indicate that the downregulation of ARHGAP18 can inhibit the proliferation of PASMCs. We then examined whether PASMC migration was affected by the downregulation of ARHGAP18 (Figure 6F). PASMCs transfected with control or siARHGAP18 for 48 h were subjected to an in vitro scratch wound assay. When ARHGAP18 was downregulated by siRNAs, the migration distance was reduced by 37.3% compared with that in the control. Moreover, the PDGF signaling-induced promotion of the migration of PASMCs was impaired when ARHGAP18 was downregulated by siRNAs. Taken together, these results indicate that endogenous ARHGAP18 is necessary to promote the proliferation and migration of PASMCs.

## 3. Discussion

PDGF signaling is implicated in the pathogenesis of vascular diseases, including PAH [29]. Considering that imatinib serves as a PDGF–receptor tyrosine kinase inhibitor, recent studies have been conducted to explore its potential as a treatment for PAH. Imatinib alleviates PDGF signaling-induced phenotypic changes, such as increased cell proliferation and migration of PASMCs [30,31,32]. Moreover, the PDGF signaling-induced upregulation of Ca(2+)-sensing receptor (CaSR) and protease-activated receptor 2 (PAR-2) expression was reduced by imatinib [33,34]. The elevated paxillin tyrosine phosphorylation in PASMCs induced by PDGF signaling was also attenuated by imatinib [35]. miRNAs have been reported as critical players in the regulation of PDGF signaling in PASMCs [36,37]. PDGF signaling can modulate the expression of specific miRNAs to regulate PASMC phenotypes, whereas certain miRNAs can regulate components of the PDGF signaling pathway [3]. Given the impact of miRNAs on PASMC function and their involvement in vascular disease pathogenesis, investigating the interplay among PASMCs, PDGF signaling, miRNAs, and imatinib is of considerable scientific interest. However, to date, the imatinib-mediated epigenetic regulation of gene expression has not been extensively evaluated in PASMCs. Our study suggests a functional relationship between imatinib-mediated miRNA regulation and the PDGF signaling-induced PASMC phenotype.

Recently, the molecular link between imatinib and miRNA expression in cancer cells has been elucidated. The expression levels of miR-150, miR-146a, and miR-142-3p changed after imatinib therapy in patients with CML [18]. Imatinib enhances the expression level of miR-30a to promote apoptosis in giant cell tumor cells and inhibits the expression of miR-483-3p to regulate mitochondrial respiratory complexes in gastrointestinal stromal tumors [38,39]. In contrast, several miRNAs affect the function of imatinib. For example, miR-144/451 contributes to acquired imatinib resistance in CML cells, whereas miR-203 and miR-199a/b-5p sensitize imatinib-resistant CML cells [22,40,41]. In this study, PDGF signaling reduced the expression of miR-335-5p while upregulating miR-409-5p, and both alterations were reversed by imatinib. This indicates that imatinib may broadly modulate PDGF-regulated miRNA expression. While our mechanistic investigation focused on the miR-335-5p–ARHGAP18 axis, these findings highlight the potential of imatinib to normalize a broader network of dysregulated miRNAs in PASMCs. Considering that miR-335-5p is essential for inhibiting the proliferation and migration of PASMCs, our study reveals that imatinib modulates PASMC function, at least in part, by regulating miR-335-5p expression. In addition, the proliferation and migration of PASMCs were inhibited by miR-335-5p to the same extent as in imatinib-treated PASMCs (Figure 4A,C). Therefore, we further propose that miRNAs, such as miR-335-5p, may mimic or enhance the therapeutic effects of imatinib.

ARHGAP18 was identified as a novel target of miR-335-5p. ARHGAP18 is a crucial factor in the regulation of Rho GTPases, which are important regulators of the cell cytoskeleton, controlling cell shape, migration, and proliferation [42]. ARHGAP18 regulates RhoA activity to control actin cytoskeleton organization, thereby promoting cell migration. The ARHGAP18-mediated suppression of RhoA activity is required for cell spreading, which is essential for promoting cell migration. In addition, ARHGAP18 is involved in the metastatic prowess of cancer cells by promoting cell proliferation, migration, and invasion [43,44,45]. Based on previous reports, a high expression level of ARHGAP18 in patients with hepatocellular carcinoma and breast cancer is correlated with a poorer survival rate [44,45]. In this study, we found that ARHGAP18 is also essential for promoting the proliferation and migration of PASMCs. Thus, PDGF signaling-induced downregulation of miR-335-5p likely contributes to PASMC proliferation and migration through upregulation of ARHGAP18. Although ARHGAP18 is known to regulate RhoA activity, we did not directly measure RhoA-GTP levels or ROCK activity in this study. Future investigations will be important to clarify the mechanistic link between the miR-335-5p–ARHGAP18 axis and RhoA/ROCK signaling.

To support our observation of ARHGAP18 involvement in the regulation of PDGF signaling-mediated PASMC phenotype, we analyzed publicly available Gene Expression Omnibus (GEO) datasets comparing PASMCs isolated from healthy donors and patients with PAH. We found that ARHGAP18 expression was 1.74-fold higher in PASMCs isolated from patients with idiopathic PAH [46] and 1.7-fold higher in PAH-derived PASMCs [47]. Together, our studies provide preliminary evidence supporting the role of the miR-335-5p–ARHGAP18 axis in the progression of vascular diseases such as PAH. At present, no clinical trials for miRNA-based therapy for PAH have been conducted. However, further investigation into imatinib-mediated miRNA regulation in PASMCs may lead to the development of novel and effective therapeutic strategies.

## 4. Materials and Methods

### 4.1. Cell Culture

Human primary pulmonary artery smooth muscle cells (PASMCs) were purchased from Lonza (Basel, Switzerland, CC-2581) and were maintained in Sm-GM2 medium (Lonza) containing 5% fetal bovine serum (FBS). For stimulation experiments, cells were treated with 40 ng/mL PDGF-BB and/or 5 µM imatinib under starvation conditions. For starvation conditions, cells were maintained in Dulbecco’s modified Eagle’s medium (DMEM, SH30243.01) containing 0.2% FBS for 16 h. Recombinant human PDGF-BB and imatinib were purchased from R&D Systems (Minneapolis, MN, USA, 220-BB) and Sigma-Aldrich (St. Louis, MO, USA, SML1027), respectively.

### 4.2. Quantitative Reverse Transcriptase-PCR (qRT-PCR)

Quantitative analysis of the change in expression levels was performed using real-time PCR. Primer sequences used were as follows: *ARHGAP18*, 5′-GAGTGCAAGCTCCCCATCTT-3′ and 5′-TGTCATCATCAAGGCAGCGT-3′, *RASA1*, 5′-CCAAACTGCCCACTTCGTTG-3′ and 5′-CAACGGTATGGCCACCTCTT-3′, *SMARCA2*, 5′-GGTTTGGGAGCACAAGCAAG-3′ and 5′-GTGCTTCTGGTCCGAACA GA-3′, *ARHGEF2*, 5′-ATCTACCCCTCCGACAGCTT-3′ and 5′-TTCCGCATGTTGAGG GAGTC-3′, and *ROCK1*, 5′-CTAACCCTGCAACTGGAGCA-3′ and 5′-TTGTAGCTCCCGCATCTGTC-3′. mRNA expression was normalized to the expression levels of *GAPDH* or 18S rRNA. For quantification of mature miRNAs, the Mir-X miRNA qRT-PCR TB Green^®^ Kit (Takara Bio Inc., Kusatsu, Shiga, Japan) was used according to the manufacturer’s instructions, and miRNA expression was normalized to U6 small nuclear RNA. Data were analyzed using the comparative C_T_ method in the Bio-Rad Manager software version 2.1 (Bio-Rad Laboratories, Hercules, CA, USA). All data represent the mean ± standard error (SE) from at least three independent experiments performed in triplicate.

### 4.3. miRNA Mimics and Anti-miRNA Oligonucleotides

Chemically modified double-stranded RNAs designed to mimic the endogenous mature miR-335-5p was purchased from Genolution Pharmaceuticals (Seoul, Republic of Korea). Antisense inhibitor RNAs (anti-miR-335-5p) and negative control oligonucleotides were obtained from Bioneer (Daejeon, Republic of Korea). The miRNA mimics and anti-miRNA oligonucleotides were transfected at 5 nM and 50 nM, respectively, using RNAiMAX (Invitrogen, Carlsbad, CA, USA) according to the manufacturer’s protocol.

### 4.4. Cell Counting Kit-8 Assay

Cell proliferation was assessed using the Cell Counting Kit-8 (Dojindo Laboratories, Kumamoto, Japan). PASMCs were seeded at 5 × 10^3^ cells/well in 96-well plates in triplicate. At four days post-transfection with miRNAs, 10 μL of CCK-8 reagent was added to each well and incubated for 1 h. Absorbance was measured at 450 nm using a SpectraMax 190 plate reader (Molecular Devices, Sunnyvale, CA, USA).

### 4.5. In Vitro Scratch Wound Assay

PASMCs transfected with miR-335-5p mimics, anti-miR-335-5p, or siARHGAP18 were plated in 6-well plates, and three scratch wounds were generated with a 200 μL disposable pipette tip. Scratch wounds were photographed after 0, 4, 8, 12, and 24 h. using a Primovert microscope stand with binocular phototube (Zeiss, Oberkochen, Germany) equipped with a digital camera (Axiocam 105 color, Zeiss), and wound widths were quantitated with ImageJ software version 1.53k (NIH, Bethesda, MD, USA).

### 4.6. Immunoblotting

Cells were lysed in TNE buffer (BR161-0737) containing 50 mM Tris–HCl (pH 7.4), 100 mM NaCl, and 0.1 mM EDTA. Total cell lysates were separated by SDS-PAGE, transferred to PVDF membranes, immunoblotted with antibodies, and visualized using an enhanced chemiluminescence detection system (Bio-Rad Laboratories, Hercules, CA, USA). The antibodies used for immunoblotting were anti-pAkt (#4060) from Cell Signaling Technology (Danvers, MA, USA), anti-ARHGAP18 (NBP2-81704) from Novus Biologicals (Cambridge, MA, USA), and anti-Akt (sc-5298) and anti-β-actin (sc-47778) from Santa Cruz Biotechnology (Dallas, TX, USA).

### 4.7. Luciferase Reporter Constructs

Two partial 3′UTR sequences of ARHGAP18 were cloned into the pIS0 vector (Addgene, Watertown, MA, USA) downstream of the luciferase gene. The predicted MRE-containing 3′UTR sequence (3′UTR #1) was amplified using 5′-ACCGAGCTCCTCACATCTACCCTTTCCCAGTT-3′ and 5′-AGTGGCCGGCCCCCTTCATTAGAGACATCAAGAGC-3′. The remaining portion of the 3′UTR that does not contain the MRE (3′UTR #2) was amplified using 5’-ATGGAGCTCGGGGAATGCTGTTAATGAGAACA-3’ and 5’-ATGGGCCGGCCTCACAGCAAAAGCAGTAGGT-3’. The MRE mutant construct for 3’UTR sequence (3′UTR #1 mut) was generated using primers, 5′-ACCGAGCTCCTCACATCTACCCTTTCCCAGTT-3′ and 5′-ATTGGCCGGCCCCCTTCATTAGAGACATGTTTCCCTTC-3′.

### 4.8. Luciferase Assay

Cos7 cells (ATCC, Manassas, VA, USA) were co-transfected with 5 nM miR-335-5p mimic or negative control and the luciferase reporter constructs using Lipofectamine 2000 (Invitrogen, Carlsbad, CA, USA). A β-galactosidase expression plasmid was used as an internal transfection control. After 24 h, luciferase activity was measured using the Luciferase Assay System (Promega, Madison, WI, USA) and quantified with a GloMax^®^ 96 Microplate Luminometer (Promega). Luciferase activity was normalized to β-galactosidase activity.

### 4.9. Statistical Analysis

For each assay, three experiments were conducted in triplicate, and data are presented as the mean with standard error. Statistical significance was determined using Student’s *t*-test, one-way ANOVA, or two-way ANOVA, as appropriate, with GraphPad Prism 8 software (GraphPad Software Inc., San Diego, CA, USA). Difference were considered statistically significant at *p* < 0.05. Significance levels are indicated as follows: *p*  <  0.05 (*), *p*  < 0.005 (**), *p*  < 0.0005 (***), and *p*  < 0.0001 (****).

### 4.10. Bioinformatic Analysis

To investigate the expression of *ARHGAP18* in PASMCs from patients with PAH, we searched the GEO database using the keyword “pulmonary arterial hypertension.” Among the available datasets, we selected GSE263226 (https://www.ncbi.nlm.nih.gov/geo/query/acc.cgi?acc=GSE263226: accessed on 28 November 2024) and GSE144932 (https://www.ncbi.nlm.nih.gov/geo/query/acc.cgi?acc=GSE144932: accessed on 9 June 2022). These datasets were downloaded from NCBI GEO and reanalyzed to assess the differential expression of *ARHGAP18* between normal and PAH conditions. The GSE144932 dataset comprises PASMCs derived from four patients with idiopathic PAH and four donor controls [46]. The GSE263226 dataset comprises PASMCs isolated from five patients with PAH and four donor controls. The expression level of *ARHGAP18* in GSE144932 was obtained using GEO2R (https://www.ncbi.nlm.nih.gov/geo/geo2r/: accessed on 9 June 2022) and normalized as transcripts per million (TPM). For GSE263226, GEO2R-based analysis was not available; therefore, raw read count data were downloaded from GEO and normalized to reads per kilobase of transcript per million mapped reads (RPKM). *ARHGAP18* expression levels were calculated for each sample, and fold changes were calculated to compare expression between PAH and control groups.

## 5. Conclusions

In this study, we found that imatinib effectively suppresses PDGF-induced proliferation and migration of PASMCs by regulating miR-335-5p, which serves as a key inhibitor of PDGF signaling and exerts antiproliferative and antimigratory effects on PASMCs. Moreover, we further identified ARHGAP18 as a novel direct target of miR-335-5p. The discovery of the imatinib–miR-335-5p–ARHGAP18 axis provides a new therapeutic opportunity for PAH through miRNA-based regulation.

## Data Availability

The original contributions presented in this study are included in the article. Further inquiries can be directed to the corresponding author.
